# Systematic analysis of ovarian cancer platinum-resistance mechanisms via text mining

**DOI:** 10.1186/s13048-020-00627-6

**Published:** 2020-03-11

**Authors:** Haixia Li, Jinghua Li, Wanli Gao, Cheng Zhen, Limin Feng

**Affiliations:** 1 Department of Obstetrics & Gynecology, Beijing TianTan Hospital, Capital Medical University, Bejing, 100050 China; 2 The Fifth Medical Center of Chinese PLA General Hospital, Beijing, 100039 China

**Keywords:** Platinum resistance, Ovarian cancer, Text mining

## Abstract

**Background:**

Platinum resistance is an important cause of clinical recurrence and death for ovarian cancer. This study tries to systematically explore the molecular mechanisms for platinum resistance in ovarian cancer and identify regulatory genes and pathways via text mining and other methods.

**Methods:**

Genes in abstracts of associated literatures were identified. Gene ontology and protein-protein interaction (PPI) network analysis were performed. Then co-occurrence between genes and ovarian cancer subtypes were carried out followed by cluster analysis.

**Results:**

Genes with highest frequencies are mostly involved in DNA repair, apoptosis, metal transport and drug detoxification, which are closely related to platinum resistance. Gene ontology analysis confirms this result. Some proteins such as TP53, HSP90, ESR1, AKT1, BRCA1, EGFR and CTNNB1 work as hub nodes in PPI network. According to cluster analysis, specific genes were highlighted in each subtype of ovarian cancer, indicating that various subtypes may have different resistance mechanisms respectively.

**Conclusions:**

Platinum resistance in ovarian cancer involves complicated signaling pathways and different subtypes may have specific mechanisms. Text mining, combined with other bio-information methods, is an effective way for systematic analysis.

## Background

Ovarian cancer is the most lethal cause of all gynecological malignancies [1]. Due to lack of specific symptoms, the majority of patients (60%) are diagnosed at advanced stages and the five-year survival rate is about 30% [2, 3]. Nowadays cytoreducitve surgery combined with chemotherapy has been accepted as a standard treatment of this disease, where platinum-based agents such as cisplatin and carboplatin are considered to be the essential components of most chemotherapy regimens [4–6]. Initial response rate to such first-line chemotherapy is as high as 65–80%. However, about half of these patients eventually develop platinum resistance, leading to an unfavorable prognosis [2]. Presently, platinum-resistance is a major obstacle in the treatment of ovarian cancer.

Although a plenty of genes and pathways have been investigated for platinum resistance in ovarian cancer, mechanisms of drug resistance are still not fully understood. Most researchers examined only a small part of genes, meanwhile the majority of them focused on specific subtypes of ovarian cancer. As platinum resistance seems to be regulated by sophisticate molecular networks, we try to systematically assess reported genes with text mining and other bioinformatics methods, quantitatively describe their relationships and make prediction of potential regulatory molecules and pathways in this study.

## Methods

The methods for data preparation and gene identification have been described previously [7]. Briefly, *Ovarian cancer AND (cisplatin OR carboplatin)* were used as retrieval statement on Pubmed and 6160 literatures were listed (up to July 24th, 2017). All abstracts were collected from PubMed retrieval system. Genes and proteins were identified with ABNER (V1.5) [8, 9] and were verified based on Entrez Gene Database. To cover the description of cisplatin and carboplatin, words and shorthands such as *“platinum”, “platin”, “cisplatin”, “DDP”, “carboplatin”* and *“CBP”* were selected*.* Similarly, both *“resistance”* and *“resistant”* were identified. Only the genes that co-appeared with these two groups of words in the same sentence will be treated. If a gene appeared several times in one sentence, it would be counted once. Word frequency analysis was performed with Microsoft Excel 2010. Gene ontology analysis was carried with FunRich (V3.0) software [10] and *p*-value were corrected with Bonferroni method.

Protein-protein interaction (PPI) network analysis was performed using Cytoscape (V3.4.0). Plugins such as BisoGenet [11] and CytoNCA [12] were used to generate network, while interaction information from MINT [13], BIND [14, 15], BioGrid [16], DIP [17], IntAct [18] and HPRD [19] were used for analysis. All interactions were based on experiments. Hierarchical cluster analysis was performed between genes and cancer subtypes (*“serous”, “mucinous”, “endometrioid”, “clear cell cancer”* or *“OCCC”*) using HemI (V1.0) [20] with maximum distance similarity metric. Data were normalized for each subtype in advance.

## Results

### Platinum-resistance related genes in ovarian cancer

According to the criterion of frequency analysis, 473 genes were identified within 6160 abstracts and top genes among them (count≥15) were listed in Table 1. *TP53* were mentioned more than 100 times, while *ABCB1, AKT1*, *ERCC1* and other genes were also widely studied in the past years.

**Table 1:** The top platinum-resistance related genes based on text mining

Gene	Description	Count
*TP53*	tumor protein p53	108
*ABCB1*	ATP binding cassette subfamily B member 1	64
*AKT1*	AKT serine/threonine kinase 1	59
*ERCC1*	ERCC excision repair 1, endonuclease non-catalytic subunit	40
*BCL2*	BCL2, apoptosis regulator	28
*EGFR*	epidermal growth factor receptor	27
*BRCA1*	BRCA1, DNA repair associated	26
*PIK3CA*	phosphatidylinositol-4,5-bisphosphate 3-kinase catalytic subunit alpha	25
*MAPK1*	mitogen-activated protein kinase 1	24
*ABCC1*	ATP binding cassette subfamily C member 1	22
*IL6*	interleukin 6	20
*NFKB1*	nuclear factor kappa B subunit 1	20
*STAT3*	signal transducer and activator of transcription 3	19
*MTOR*	mechanistic target of rapamycin kinase	18
*PARP1*	poly (ADP-ribose) polymerase 1	17
*TNFSF10*	TNF superfamily member 10	17
*BRCA2*	BRCA2, DNA repair associated	15
*HDAC1*	histone deacetylase 1	15
*TNF*	tumor necrosis factor	15

Only the genes that co-appeared with drug name (such as “cisplatin”) and phenomenons (such as “resistance”) in the same sentence will & be treated

### Gene ontology analysis

To explore the functions of these genes, gene ontology (GO) analysis was carried out. Significant biological processes that may involve (corrected *p* < 0.05) in platinum resistance were shown in Table 2. Apoptosis were highlighted as the most significant process, while signal transduction, cell communication, cell cycle, anti-apoptosis, and nucleobase & nucleic acid metabolism were also included.

**Table 2:** Significant biological process (GO analysis) for platinum resistance in ovarian cancer

Biological Process	Number of Genes	Corrected *P*-Value
Apoptosis	31	6.64 × 10^− 13^
Signal transduction	154	5.19 × 10^−08^
Cell communication	143	9.82 × 10^−07^
Regulation of cell cycle	8	0.012
Anti-apoptosis	6	0.020
Regulation of nucleobase, nucleoside, nucleotide and nucleic acid metabolism	99	0.024

All 473 identified genes were treated as input. *P* values were corrected with Bonferroni method

### PPI network analysis

**Fig. 1:**
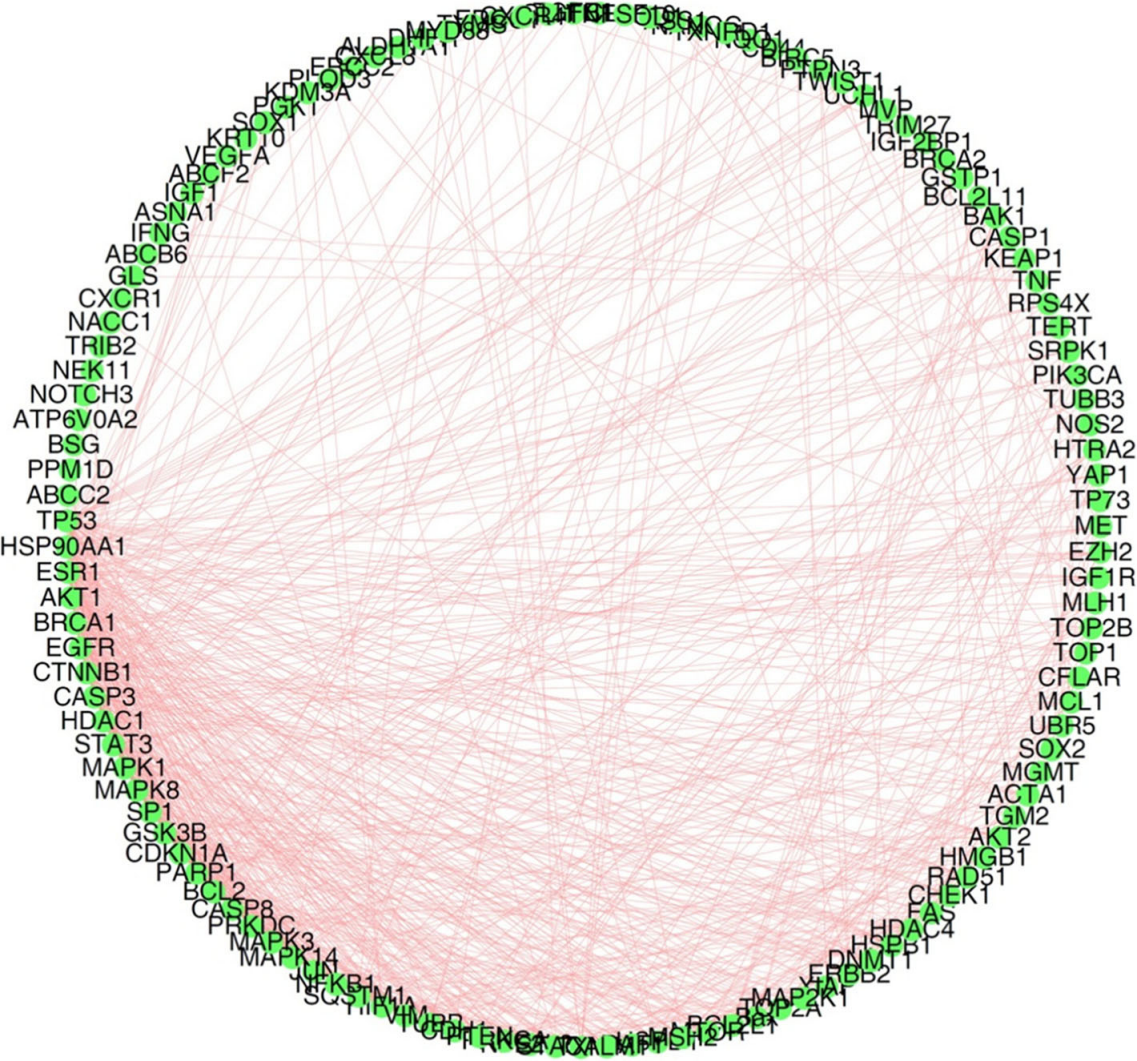
The PPI network of platinum-resistance related genes. Self-loops and isolated nodes were deleted. All interactions were based on experiments. Network was generated just among input nodes rather than their neighbours. Molecules with count less than 3 were excluded before PPI analysis

To find out important molecules in platinum resistance mechanism, PPI network was generated with Cytoscape (V3.4.0) software and its plugins. The interactions were illustrated in Fig. 1 and the most popular nodes with their degrees (the number of interactions) were listed in Table 3. TP53 has the highest degree than other proteins, which implies the critical function of it in platinum resistance regulation. In addition, HSP90AA1 (degree = 41), ESR1 (degree = 40), AKT1 (degree = 39), BRCA1 (degree = 35) and other proteins were also predicted as remarkable hubs among the signaling network.

**Table 3:** The top nodes (degree> 20) in platinum-resistance related PPI network

Node	Description	Degree
TP53	tumor protein p53	56
HSP90AA1	heat shock protein 90 alpha family class A member 1	41
ESR1	estrogen receptor 1	40
AKT1	AKT serine/threonine kinase 1	39
BRCA1	BRCA1, DNA repair associated	35
EGFR	epidermal growth factor receptor	34
CTNNB1	catenin beta 1	31
CASP3	caspase 3	30
HDAC1	histone deacetylase 1	28
MAPK1	mitogen-activated protein kinase 1	26
STAT3	signal transducer and activator of transcription 3	26
MAPK8	mitogen-activated protein kinase 8	25
SP1	Sp1 transcription factor	24
GSK3B	glycogen synthase kinase 3 beta	23
CDKN1A	cyclin dependent kinase inhibitor 1A	23
PARP1	poly (ADP-ribose) polymerase 1	22
BCL2	BCL2, apoptosis regulator	21
CASP8	caspase 8	21

All edges are treated as undirected. The degree of each node is calculated with CytoNCA, a plugin for Cytoscape

### Cluster analysis for subtypes

Based on histopathology, ovarian cancer can be mainly classified into four subtypes: serous, mucinous, endometrioid and ovarian cancer of clear cell (OCCC) [21]. Each major histological type has characteristic morphological features and biological behaviors [22], and the incidence of platinum resistance differs from the others. For example, mucinous ovarian cancer has been reported to have a much lower sensitivity and higher resistance rate compared with serous ovarian cancer [23, 24].

**Fig. 2:**
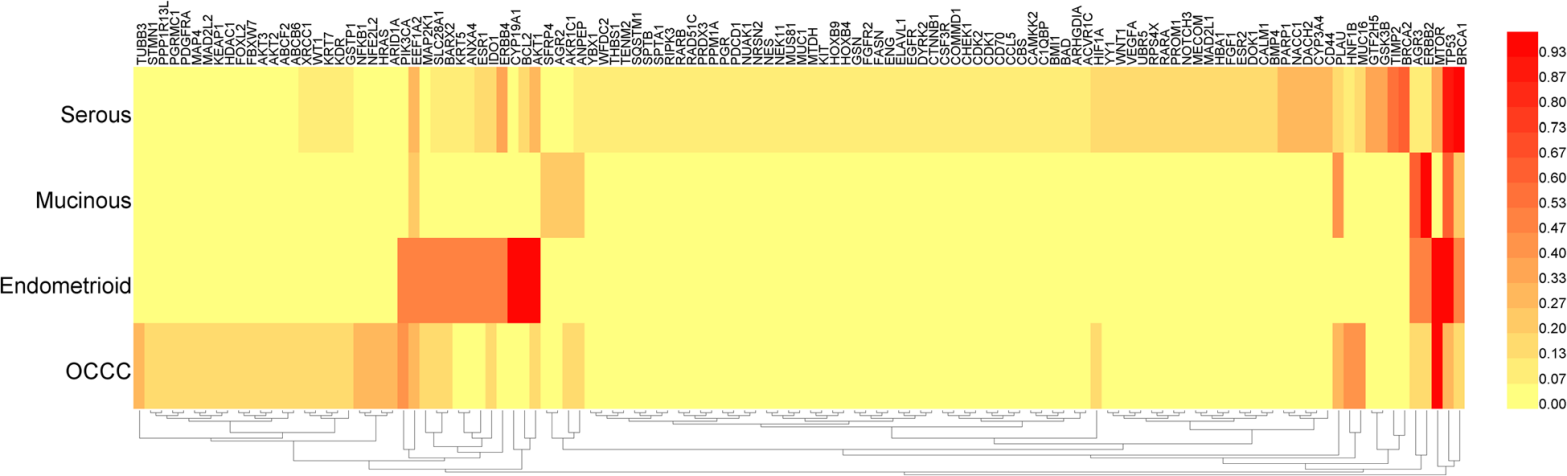
Hierarchical cluster analysis for genes among subtypes of ovarian cancer. Cluster analysis was performed based on maximum-linkage, using similarity metric of maximum distance. Each subtype was normalized respectively before cluster analysis

To investigate the specific regulatory molecules for each subtype, genes co-appearing with “*serous*”, “*mucinous*”, “*endometrioid*” and “*clear cell*” (or *OCCC*) were collected respectively, then cluster analysis were performed. As shown in Fig. 2, each subtype has its distinctive combination for platinum-resistance molecules. Some genes such as *TP53* are commonly focused in most subtypes. By comparison, *BCL2* and *AKT1* were frequently mentioned in endometrioid cancer while *ERBB2* and *AGR3* were repeatedly mentioned in mucinous cancer. Such genes may be regarded as specific regulators or markers for each subtype.

## Discussion

Cisplatin and carboplatin exert antitumor effects by binding to DNA and forming cross-links, thus disrupts DNA structure and finally results in cell apoptosis [25]. Dysregulation in that process may cause platinum resistance. Among all possible regulatory mechanisms, the most important ones include the followings [26]: (1) Suppressed uptake or enhanced efflux can reduce cytosol accumulation of platinum. (2) Drug detoxification mechanism can protect cells from bioactive platinum aquo-complexes. (3) DNA repair can be activated and enhanced to restore DNA damages. (4) Changes in signaling pathways make cells evade fate of apoptosis. These mechanisms and pathways interact with each other, making platinum-resistance regulation very complex. It should be noted that cisplatin and carboplatin share similar molecular structures and are cross-resistant in most cases. In contrast, oxaliplatin are not cross-resistant with them, which may be explained by the lipophilic cyclohexane residue [27]. So oxaliplatin resistance is not discussed in this study.

According to Table 1, most of the top genes can be classified into the four categories mentioned above, and apoptosis is the most significant process in Table 2. The tumor-supressor P53 is a central hub for the activation of intrinsic apoptotic pathway [28]. It can trigger cell death via the expression of apoptotic genes and by inhibiting the expression of anti-apoptotic genes [29]. *BCL2* can inhibit cell death induced by cytotoxic factors such as chemotherapeutic drugs and enhance cell resistance [30, 31].

For platinum accumulation, both *ABCB1* (MDR1) and *ABCC1* (MRP1) belong to ATP binding cassette (ABC) transport protein family, which works as ATP-dependent drug efflux pump and is responsible for decreased platinum accumulation [32, 33]. Among all the identified molecules, *ABCG2* (count = 13) and *ABCC2* (count = 10) have similar functions though not listed in Table 1. Another example for transporter protein is *SLC31A1* [34] (also known as *CTR1*), a member of copper transporter family, which plays a significant role in platinum uptake [35].

For DNA damage/repair, *ERCC1* (ERCC excision repair 1) is a critical member of nucleotide excision repair induced by platinum [36]. Meanwhile, *BRCA1* [37] and *BRCA2* [38] exert their functions in double-stranded breaks repair of DNA. *PARP1* can recognize DNA lesions and modifies various nuclear proteins which are involved in the regulation of DNA repair [39].

Both *GSTA1* (count = 12) and *GSTP1* (count = 9) belong to the top 10% of all identified genes though not listed in Table 1. The expression products of them are members of cellular detoxification system, which can add glutathione to platinum, block the formation of Pt-DNA and reduce cytotoxicity of platinum [40, 41].

Besides, some popular genes such as *AKT1*, *EGFR*, *PIK3CA*, *MAPK1*, *NFKB1* and *MTOR*, are difficult to be classified. All of them have multiple functions in physiological and pathological processes and are regarded as key nodes in platinum-resistance signaling network (as shown in Table 3). Their effects toward platinum resistance have been extensively explored, together with their various targets or regulators [42–45].

There are specific genomic alterations and gene-expression patterns for different subtypes of ovarian cancer. According to previous reports, *K-RAS* mutation is very common in mucinous ovarian carcinomas (75%), but the rate is generally low in clear cell carcinomas [46, 47]. Meanwhile, genes involved in nucleotide excision repair (such as *XPB* and *ERCC1*), were found to be preferentially expressed in ovarian clear cell carcinomas [48, 49]. That suggests each subtype may have specific mechanism and molecular character for platinum resistance, but there are few reports for this topic. In our study, genes were enriched according to their co-occurring subtypes and then subjected to cluster analysis. This method helps us understand the differences in regulatory mechanisms among subtypes of ovarian cancer. It is also meaningful for clinical accurate diagnosis and individualized treatment of ovarian cancer.

A potential limitation in this study is the performance of text mining. It can recognize names of genes and proteins, calculate their frequencies and judge the functions of them via co-occurrence analysis, but it cannot really “understand” literatures. However, it is still an effective method to quantitatively assess gene functions and their relationships, especially for comprehensive analysis with large input data.

## Data Availability

All data generated or analyzed in this study are included in this article.
